# Prognostic Values of Thalamic Metabolic Abnormalities in Children with Epilepsy

**DOI:** 10.3390/diagnostics15151865

**Published:** 2025-07-25

**Authors:** Farshid Gheisari, Amer Shammas, Eman Marie, Afsaneh Amirabadi, Nicholas A. Shkumat, Niloufar Ebrahimi, Reza Vali

**Affiliations:** 1 Nuclear Medicine Department, Ionizing and Non-Ionizing Radiation Protection Research Center (INIR-PRC), University of Medical Sciences, Shiraz P.O. Box 7134786916, Iran; fgheisari@gmail.com; 2 Department of Diagnostic and Interventional Radiology, The Hospital for Sick Children, University of Toronto, Toronto, ON M5G 1X8, Canada; amer.shammas@sickkids.ca (A.S.); afsaneh.amirabadi@sickkids.ca (A.A.); nicholas.shkumat@sickkids.ca (N.A.S.); 3 Department of Medical Imaging, McMaster University Hamilton, Ontario, ON L8S 2A5, Canada; eezzatmarie@gmail.com; 4 Department of Medicine, Division of Nephrology, Loma Linda University Medical Center, Loma Linda, CA 92354, USA; nebrahimi@llu.edu

**Keywords:** pediatric epilepsy, thalamic hypometabolism, refractory seizures, FDG-PET biomarkers, epilepsy surgery outcome, thalamocortical network, subcortical metabolism, quantitative PET imaging, neuroimaging prognostic marker, artificial intelligence in epilepsy

## Abstract

**Background**: Hypometabolism of the thalamus has been reported in epilepsy patients. This study aimed to investigate the prognostic value of thalamic metabolic activity in children with epilepsy. **Methods**: A total of 200 children with epilepsy and 237 children without epilepsy (sex- and age-matched control group) underwent 18F-FDG PET/CT in this study. Localization of the interictal hypometabolic epileptic focus was performed visually. Bilateral thalamic metabolic activity was evaluated qualitatively (thalamic FDG uptake in relation to the cerebral cortex) and semi-quantitatively (SUV max, normalized SUV (ratio to ipsilateral cerebellum), and absolute asymmetric index (AAI). **Results**: A total of 133 patients (66.5%) with epilepsy showed cerebral cortical hypometabolism in the interictal 18F-FDG PET study; there were 76 patients on the right side, 55 patients on the left side, and two patients on both sides. Of these 133 patients, 45 also had visually observed asymmetric hypometabolism in the thalamus. Semi-quantitatively, asymmetry was more prominent in epileptic patients. AAI was a more sensitive variable than other variables. Average AAIs were 3.89% and 7.36% in the control and epilepsy patients, respectively. Metabolic activity in the thalami was significantly reduced in epileptic patients compared to the control group. Associated hypometabolism of the ipsilateral thalamus was observed in 66.5% of epileptic patients with a focal cortical defect semi-quantitatively. Overall, 61 out of 200 patients showed thalamus hypometabolism. Some 51 out of 61 patients (83.6%) with thalamus hypometabolism showed refractory disease; however, the refractory disease was noted in 90 out of 139 (64.7%) patients without thalamus hypometabolism. Brain surgery was performed in 86 epileptic patients (43%). Some 35 out of 86 patients had thalamus hypometabolism. Recurrence of epilepsy was observed more in patients with thalamus hypometabolism (48% vs. 25%), with *p* ≤ 0.01. **Conclusion**: This study suggests that patients with thalamus metabolic abnormalities may be more medically resistant to therapy and less responsive to surgical treatments. Therefore, the thalamus metabolic abnormality could be used as a prognostic sign in pediatric epilepsy. Recent studies have also suggested that incorporating thalamic metabolic data into clinical workflows may improve the stratification of treatment-resistant epilepsy in children.

## 1. Introduction

The thalamus is a subcortical grey matter structure with hub-like information exchanges to the cerebral cortex [1]. It has some nuclei divided into anterior, medial, and lateral groups [2]. Every sensory system, except for olfaction, has a thalamic nucleus that receives, processes and delivers information to the relevant cortical area [3]. Thalamic nuclei are essential regulators of arousal, awareness, and activity [4]. Stroke in the thalamus can produce akinetic mutism, oculomotor difficulties, and thalamic pain syndrome [5].

Uptake of the analog of glucose 18F-fluorodeoxyglucose (18F-FDG) is linked to tissue metabolism [6]. It was first described in the 1970s by Brookhaven National Laboratory [7]. Moreover, 18F-FDG is taken up by high-glucose-using cells like the brain, brown adipocytes, and cancer cells; 18F-FDG is primarily used in oncology to image malignancies, and 18F-FDG uptake is measured semi-quantitatively using the Standardized Uptake Value (SUV). SUV is directly related to tumor grading. Furthermore, 18F-FDG PET can be used to diagnose, stage, and assess cancer treatment [8]. According to studies, 18F-FDG PET significantly improved the prognosis for epilepsy patients, which is primarily due to better identification of the hypometabolism epileptogenic zone [9]. Epilepsy is one of the most common neurologic disorders, affecting almost 1–2% of the population [10]. Medical treatment usually controls seizures in most patients. However, nearly 25% of pediatric patients with epilepsy become medically intractable and may require surgery. Surgery includes the local resection of the epileptogenic focus. In clinical practice, resection of epileptic focus might still fail to control the seizures in some patients due to the following:

- Incomplete resection of the epileptogenic zone;
- Complex epileptogenic network, which may involve the subcortical structures.

The thalamus is one of the potential critical components in the subcortical neuronal circuits that may take part in the initiation and spread of seizures. A debate about the relative importance of the thalamus and cortex in the pathogenesis of seizures began in the 1800s and continues today [11]. Cortical hypometabolism is a characteristic finding for a cortical epileptogenic focus in the interictal 18F-FDG PET study. However, subcortical metabolic changes in the thalamus in epileptic patients have not been well described. When 18F-FDG is administered in the interictal phase, hypometabolism is demonstrated in the epileptogenic zone [11]. Several diagnostic methods have been performed to delineate the epileptogenic zones, and researchers have attempted to discover whether such studies can predict postoperative seizure outcomes. Some patients with focal epilepsy also show hypometabolism in the thalamus, though the significance of this finding is not well evaluated. Focal seizures refractory to medical therapies can often be treated successfully with surgical resection of the lesion in selected cases. Thalamic hypometabolism has been well reported in people with focal epilepsy [12]. The thalamus’ role in seizure activity is likely to be complicated because of its diffuse connections across the brain. The complex dynamics of thalamocortical loops—particularly involving reciprocal excitatory and inhibitory pathways—have been implicated in the maintenance of seizure persistence and resistance to pharmacologic treatment. Aberrant thalamocortical synchrony may act as a stabilizing feedback loop that perpetuates pathological neural firing, which is especially relevant in medically refractory pediatric epilepsy.

This study aimed to investigate the prevalence of thalamus metabolic abnormality and how thalamic hypometabolism affects prognosis in patients with focal epilepsy in a large sample of patients. In the context of epilepsy pathophysiology, the thalamus is now increasingly viewed as not only a relay station but also a dynamic integrator of epileptiform discharges. This evolving view suggests that the thalamus actively contributes to seizure propagation via both direct corticothalamic relays and diffuse modulatory pathways, highlighting its role beyond passive signal relay and emphasizing its prognostic importance in drug-resistant epileptic circuits.

Recent neurodevelopmental studies suggest that thalamocortical feedback loops may influence the transition from interictal spikes to clinical seizures, particularly in childhood epilepsy syndromes [13].

This may be due to the unique biophysical properties of thalamic relay cells, which are capable of generating burst-mode firing when hyperpolarized, thereby promoting rhythmic epileptic activity in susceptible networks.

In addition, metabolic dysregulation within thalamic nuclei—especially the mediodorsal and pulvinar regions—has been associated with cognitive impairment in epilepsy, indicating a broader functional impact [14].

Functional imaging studies have demonstrated that abnormalities in thalamic metabolism can emerge even in the early stages of epileptogenesis, especially in genetically predisposed pediatric populations. The interplay between thalamocortical circuits and hippocampal connectivity may explain why seizures persist in some patients despite cortical lesion resection.

**Figure 1:**
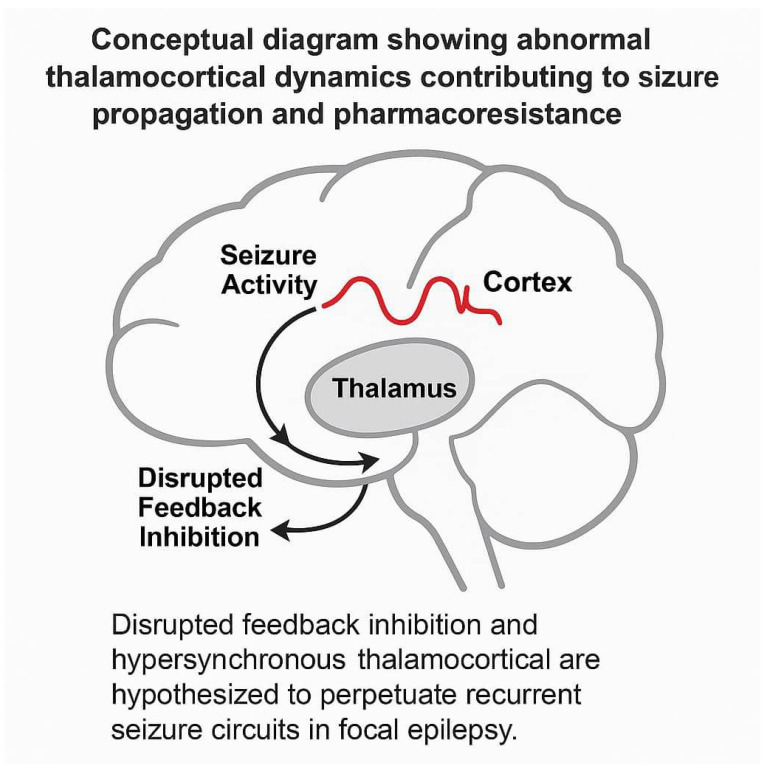
Conceptual diagram showing abnormal thalamocortical dynamics contributing to seizure propagation and pharmacoresistance. Disrupted feedback inhibition and hypersynchronous thalamocortical firing are hypothesized to perpetuate recurrent seizure circuits in focal epilepsy.

Moreover, thalamic pathology may not be detected by conventional MRI, making PET-based metabolic imaging a valuable complementary modality [15]. A schematic diagram illustrating the hypothesized role of thalamocortical loops in pharmacoresistant epilepsy is provided in Figure 1 for clarity.

## 2. Materials and Methods

This study was conducted in accordance with the Declaration of Helsinki and approved by the hospital Research Ethics Board (REB# 1000078477, date of approval 24 January 2022). Patient consent was waived due to the retrospective nature of the study. In this study, 237 children without epilepsy (control group) (146 boys and 91 girls with a mean age of 10 ± 3.8 years) and 200 children with epilepsy (case group) (106 boys and 94 girls with a mean age of 9 ± 3.6 years) were subjected to interictal 18F-FDG PET/CT between 2015 and 2021. Detailed clinical profiles were documented for all patients, including the age of epilepsy onset (mean: 3.2 years; range: 6 months to 9 years), confirmed diagnosis based on ILAE criteria, and neuroimaging evidence of structural abnormalities localized to temporal, frontal, or parietal cortices.

All patients in the epilepsy group had received multiple antiepileptic drugs (AEDs) and had been categorized by failure to achieve sustained seizure freedom; 18F-FDG PET was performed as a pre-surgical workup evaluation. The patients were called pharmacoresistant when adequate trials of two or more anti-seizure medications had been used with sufficient dosage and the medications had been adhered to. The control group was selected from patients who had malignancy with no head and neck abnormality.

Exclusion criteria for the cases were a history of previous brain surgery for other reasons except for epilepsy and a follow-up period of less than one year after 18F-FDG PET/CT results if patients were treated surgically to assess treatment outcome. The control group was selected from oncologic patients who were typically developing normal healthy children with normal brain MRI (if present) and no prior history of brain surgery or any medical, developmental, or psychiatric disorders. Recent advances in pediatric neuroimaging protocols have standardized interictal PET acquisition to minimize variability in metabolic assessment [16].

### 2.1. Imaging Procedure

An intravenous catheter was placed, under local anesthetic, for PET imaging. In this study, patients had their eyes open and ears non-occluded, while noise and light were minimal. The FDG was administered intravenously at 3.7 MBq/kg [9]. Approximately 45 min after the administration of FDG, PET imaging started. Two PET/CT cameras were used in this work. Studies between 2015 and 2017 were completed on a Philips Gemini GXL (Philips Healthcare, Best, The Netherlands) with a matrix size of 128 × 128, at 2 mm slice thickness and 10 min per acquisition. Those acquired between 2018 and 2021 used a GE Discovery MI (GE Healthcare, Waukesha, WI, USA) with a reconstructed matrix size of 384 × 384, with 2.78 mm slice thickness and 5 min per acquisition. There was no statistically significant difference between the two subgroups. All studies were acquired in 3D mode with a 15 cm field of view and reconstructed using ordered subset expectation maximization (OSEM) iterative techniques. CT was performed for attenuation correction. Throughout the study, the head was fixed, and a technologist ensured it was in the correct position. Advances in PET technology since 2020, such as improved time-of-flight resolution and motion correction, have significantly improved image quality in pediatric cohorts [17].

### 2.2. Imaging Interpretation

**Figure 2:**
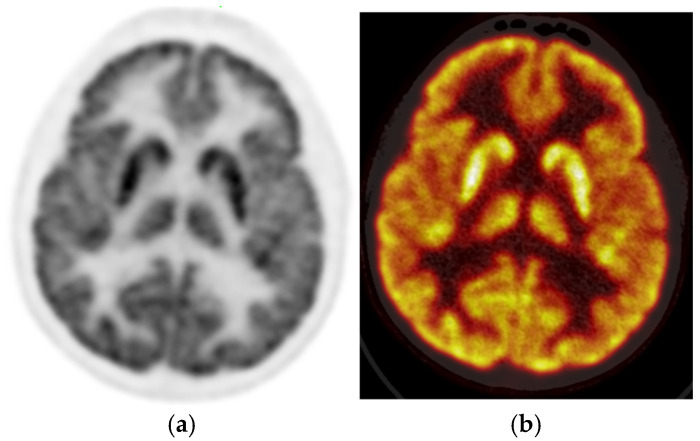
Brain 18F-FDG PET, (**a**) (sagittal grey scale) and (**b**) (sagittal rainbow color) in a 16-year-old male with no definite cortical abnormality. The uptake of the thalami is also symmetric.

**Figure 3:**
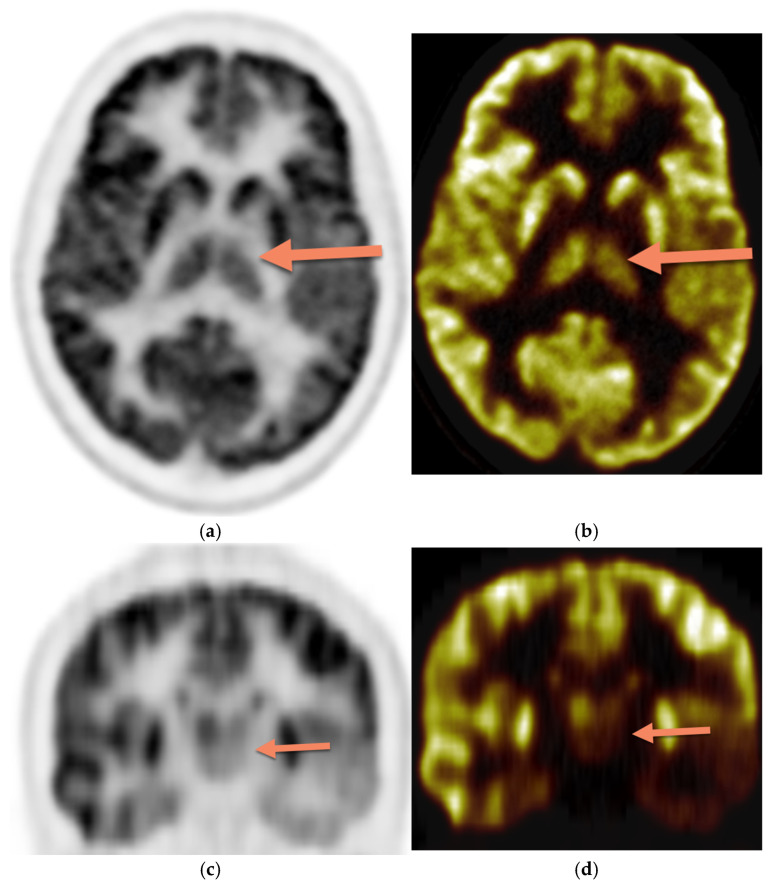
Brain 18F-FDG PET, (**a**) (sagittal grey scale), (**b**) (sagittal rainbow color), (**c**) (coronal grey scale), and (**d**) (coronal rainbow color) in a 13-year-old female with asymmetric decreased activity in the left temporal lobe. The uptake of the left thalamus is also reduced (arrow) compared with the contralateral side.

The PET images were reviewed and interpreted by two expert nuclear medicine physicians who were blinded; if there was any discrepancy, there was a consensus. Visual analysis was used to identify the location of the hypometabolic epileptogenic zone compared to the cortical activities. The metabolic activity of the bilateral thalamus was assessed qualitatively (thalamic 18F-FDG uptake in relation to the cerebral cortex and contralateral thalamus). Normal thalami are symmetric with metabolic activity similar to the normal cortex (Figure 2). Figure 3 demonstrates asymmetric decreased activity in the left temporal lobe as well as reduced left thalamus uptake.

The use of blinded consensus reading enhances interpretive reliability, which is critical for clinical PET validation in epilepsy surgery planning.

The metabolic activity of the bilateral thalamus was also assessed semi-quantitatively using SUV max, normalized SUV max (ratio to the ipsilateral cerebellum), and the absolute asymmetric index (AAI), which was calculated as 100 × [right − left]/[1/2 × (right + left)]. SUV max was used in this study as it is less observer-dependent and shows good reproducibility [18]. Since there were no definite cut-off values for normalized SUV max and AAI, the age- and sex-matched control group was used as the reference group. In the patients’ group, two standard deviation differences in AAI with age- and sex-matched controls were considered abnormal. The level of thalamic metabolism was visually categorized as hypermetabolic, hypometabolic, and normal. Recent pediatric studies have applied machine learning models to interpret SUV patterns and asymmetry scores in thalamic regions to predict seizure outcomes [18].

The correlation of the thalamic metabolic activity was made with the cerebral defect in the 18F-FDG brain scan, the clinical course of the disease (medically refractory disease), and surgical outcome at follow-up oneyear post-surgery. Patients were assessed for postoperative seizures at regular intervals for the first year following surgery and classified as seizure-free or not.

### 2.3. Image Analysis

For image analysis, all three planes were evaluated. The temporal, frontal, parietal, and occipital lobes and the thalami were all examined qualitatively. The thalamus activity was considered abnormal if there was asymmetric activity upon qualitative analysis or reduced activity upon semiquantitative analysis.

### 2.4. Statistical Analysis

Demographic variables were summarized using descriptive statistics. Continuously scaled variables were reported using means and standard deviations. Categorical variables were declared using proportions and ratios. More recent studies prefer R or Python-based platforms with statistical learning modules for PET-based prognostic analysis in pediatric epilepsy [19]. Group comparisons for continuous variables (such as SUV values and age) were performed using an independent samples *t*-test or Mann–Whitney U test depending on normality (assessed via the Shapiro–Wilk test). Categorical variables (e.g., sex, seizure type) were analyzed using chi-square or Fisher’s exact tests as appropriate. Correlation analyses between thalamic SUV and clinical features (e.g., seizure frequency, duration of epilepsy) were conducted using Pearson or Spearman coefficients based on distribution. Statistical analysis was performed using Excel version 2019, and significance was set at *p* < 0.05 (two-tailed).

## 3. Results

The study included 200 patients (94 girls and 106 boys; mean age 9 ± 3.6 years) and 237 age- and sex-matched controls (91 girls and 146 boys; mean age 10 ± 3.8 years). Patients had significantly lower mean SUV max than controls for both the right and left thalamus (7.5 ± 2.2 vs. 9.3 ± 3.6 and 7.4 ± 2.1 vs. 9.1 ± 3.6, respectively, both *p*-values < 0.05). PET was abnormal visually (cortical defect) in 133 patients (66.5%) and showed normal symmetric metabolism in the thalamus in all controls in visual analysis. Two patients (1%) had bilateral cortical hypometabolism, and both had tuberous sclerosis. In three patients (1.5%), thalamic hypometabolism was the only finding on PET. This unique subset with isolated thalamic hypometabolism may represent early or atypical epileptogenic pathways, warranting further electrophysiological correlation. In subgroup analysis based on the presumed epileptic focus, the reduction in thalamic FDG uptake was more prominent among patients with temporal lobe epilepsy compared to those with frontal lobe epilepsy (mean SUV difference = 0.21), although this finding did not reach statistical significance.

AAI was the most sensitive variable compared to the visual analysis, SUV max of thalami, and normalized SUV. Based on AAI, 61 (30%) of 200 patients were noted to have thalamic hypometabolism compared to the control group. Of these 61 patients, 45 also had thalamic hypometabolism on visual analysis. The laterality of thalamic hypometabolism was congruent with the reported side of epileptic focus on PET imaging in all the patients. The mean SUV max for the thalamus on the epileptogenic side was lower than the contralateral side in the SUV-based analysis (normalized SUV max of 1.2 vs. 1.29), which was statistically significant (*p* ≤ 0.05). All patients with thalamic hypometabolism on the epileptic side had a lower SUV max on the same side than the opposite side, demonstrating a high correlation between the visual and SUV-based analyses. Frontal hypometabolic lesions showed less correlation with thalamic hypometabolism; 4 out of 30 patients (13.3%) with frontal hypometabolic lesions on 18F-FDG PET demonstrated thalamus hypometabolism. In contrast, the highest correlation was found with the hypometabolic cortical lesions located at the temporal lobe, which can be seen in 29 out of 55 patients (52.7%) with temporal lobe defects (*p* ≤ 0.0001). Recent multimodal studies confirm this association, showing strong connectivity between the temporal cortex and thalamus in focal epilepsy using DTI and PET-MR co-registration [20]. The summary of data analysis for semiquantitative data is shown in Table 1.

Some 61 out of 200 patients demonstrated thalamus hypometabolism compared to age and sex-matched controls, and 51 out of 61 patients revealed evidence of medically intractable disease (83.6%). Meanwhile, 90 out of 139 patients with normal thalamus activity demonstrated evidence of medically refractory disease (64.7%). Therefore, medically intractable disease was more common in patients with thalamus hypometabolism (*p* ≤ 0.003) (Table 2).

Some 86 (43%) of the 200 patients were treated with surgical excision of the cortical lesion, and 30 out of 86 patients demonstrated no response to therapy and experienced continuous seizures (34.8%); the most common pathology in these patients was focal cortical dysplasia (50%). A total of 35 out of 86 patients had thalamic hypometabolism in the 18F-FDG PET study. Of 35 patients with thalamus hypometabolism, 17 demonstrated continuous seizures post-surgically (48%). Of 51/86 patients with no thalamus hypometabolism, 13 showed persistent seizures post-surgically (25%). Therefore, the response to surgery was significantly lower in patients with thalamus hypometabolism than in patients without thalamus hypometabolism (*p* ≤ 0.01). (Table 3). These findings support the prognostic value of preoperative thalamic PET patterns in surgical outcome prediction.

## 4. Discussion

This study showed a higher prevalence of thalamic hypometabolism in children with epilepsy compared to the control group cases. As the thalamus has solid reciprocal connections to all parts of the cortex and its natural tendency is to fire rhythmic bursts of the action potential, it appears reasonable to suggest that it plays a crucial role in epileptic seizure networks [21]. Widespread network interactions between cortical and subcortical regions are involved in epileptic seizures. Although the cortex is frequently emphasized as the epileptic seizure source, increasing evidence indicates that subcortical areas play an essential role in the behavioral symptoms, propagation, and, in some circumstances, initiation of epileptic seizures [22]. For example, in absence-type epilepsy in children, studies demonstrated the involvement and possibly even initiation of 3-Hz spike-and-wave attacks in the thalamus [23,24]. The thalamus and brain stem are thought to play a role in controlling cortical excitability and, as a result, seizure threshold. The basal ganglia are involved in the ictal dystonia phenomena. The cerebellum also has a role in reducing seizure frequency [25]. Recent longitudinal studies reinforce the concept that thalamic hypometabolism may precede cortical structural abnormalities, supporting its role as an early biomarker in epilepsy. This observation aligns with previous reports suggesting stronger thalamocortical connectivity in temporal circuits compared to frontal regions, which may account for the more robust predictive value of thalamic hypometabolism in temporal lobe epilepsy.

Moreover, the development of non-invasive imaging biomarkers is reshaping epilepsy diagnosis by focusing on deep brain structures like the thalamus.

Altered thalamic functional profiles have been postulated as imaging indicators of active secondary generalization in focal epilepsy [26]. The thalamus may become involved in generalized-type seizures early or late in the seizure, but once it does, it leads to the cortex. This study showed less correlation between thalamus hypometabolism and frontal cortical lesions than other cortical lesions. In human frontal seizures, the thalamus becomes involved late in the seizure and lags behind the cortex once it does [27]. The high prevalence of thalamic hypometabolism in patients with temporal lobe involvement supports the concept of secondary thalamic deactivation in prolonged focal seizures. This phenomenon, sometimes referred to as “diaschisis,” involves functional suppression in regions connected to but anatomically distant from the seizure focus [28].

In young brains, the thalamus may be more vulnerable to these trans-synaptic effects due to plasticity and ongoing myelination. Several recent pediatric studies have used voxel-based PET analysis and tractography to map such connections, highlighting altered connectivity between the thalamus and both limbic and association cortices. Furthermore, newer imaging paradigms incorporating diffusion-weighted PET and PET-MRI fusion are beginning to elucidate regional thalamic vulnerabilities based on seizure semiology.

For instance, patients with gelastic seizures often show hypometabolism in the medial thalamus, while those with absence epilepsy show diffuse bilateral thalamic deactivation. These emerging patterns suggest that different thalamic nuclei may have syndrome-specific metabolic profiles, opening new avenues for subregional targeting in treatment-resistant epilepsy [29].

Furthermore, independent focal unilateral epileptiform discharges localized to thalamic structures suggest that the thalamic centromedian nucleus can be the autonomous epileptogenic focus. The thalamus may also be responsible for sustaining ictal discharge rhythmicity by rhythmic thalamocortical loops [30]. The presence of frequent bilateral independent discharges localized to the thalamus suggests that the thalamus has some epileptogenic potential and could even be the primary source of seizures [19]. We had three (1.5%) patients for whom thalamic hypometabolism was the only finding on PET. While the specific cause of isolated thalamic hypometabolism is unknown, it could be linked to the thalamus’s primary role in initiating, propagating, and regulating seizures in various epileptic diseases [31]. Artificial intelligence models trained on metabolic brain data have recently been shown to reliably predict seizure outcomes, especially when thalamic features are included. In a growing number of clinical PET studies, thalamic metabolic metrics—especially asymmetry and regional SUV ratios—are being evaluated as potential inclusion criteria for surgical candidacy in pediatric epilepsy.

Studies have shown that ictal activity could damage the thalamus directly [21]. In this study, we found that thalamic metabolic abnormality in children with epilepsy was associated with a higher risk of refractory medication disease (intractable disease) and a higher rate of postoperative seizures. Therefore, thalamic hypometabolism may have prognostic significance in patients with epilepsy. This strengthens the notion that thalamic dysfunction is not merely a downstream effect of cortical seizures but an active contributor to seizure persistence and treatment resistance. Our study has some limitations, as it is a retrospective study, and patients and control groups were scanned using slightly different image acquisition techniques, which might slightly challenge their direct comparison, and tumor burden in the control group may have a minor impact on the measured SUV max. Moreover, imaging was performed with two different PET scanners, which may have had a minor impact on the patient’s data. PET was also not acquired in combination with an EEG. However, our sample size, use of both qualitative and quantitative PET analysis, and strict matching of control groups enhance the credibility of our findings [32]. Finally, we did not compare our results with the structural MRI findings, EEG patterns, antiepileptic medications and dosages, therapeutic plasma levels, seizure types (focal vs. generalized), and frequency. These are important factors that can be investigated in future studies.

## 5. Conclusions

Patients with epilepsy had a lower thalamic metabolic activity than the control group and on the epileptogenic side compared with the contralateral side. Thalamic hypometabolism may also help to localize the seizure focus, combined with electroclinical and other imaging modalities, in patients who do not have interictal cortical hypometabolism. Patients with thalamus metabolic abnormalities are significantly more medically resistant to therapy and less responsive to surgical treatments than patients without thalamus metabolic abnormalities. Therefore, thalamus metabolic abnormality could be used as a prognostic sign in children with epilepsy. Given the complex and dynamic role of the thalamus, future interventional strategies might include targeting thalamic nuclei via neuromodulation or tailored resection, particularly in MRI-negative epilepsy cases. There is increasing interest in stereotactic laser ablation and deep brain stimulation of the anterior or centromedian thalamic nuclei as adjuncts to standard resective surgery. Such approaches are still under evaluation but hold promise, especially for patients with bilateral cortical abnormalities or ambiguous seizure onset zones. Establishing metabolic reference ranges for pediatric thalamus via age-stratified FDG-PET data could also enhance diagnostic precision in presurgical evaluation. Moreover, AI-assisted interpretation of thalamic PET features—such as asymmetry, nodal entropy, and normalized SUV—may soon offer predictive analytics to personalize treatment plans. A multidimensional approach that combines PET, EEG, genetics, and neurocognition could further refine risk stratification and optimize surgical candidacy.

## Figures and Tables

**Table 1:** Summary of quantitative data analysis for semiquantitative data.

Thalamus Semiquantitative Analysis	Epileptic Group *n* = 200	Control Group *n* = 237	Statistical Analysis *p*-Value
SUV max	Average ± SD RT: 7.5 ± 2.2 LT: 7.4 ± 2.1	Average ± SD RT: 9.3 ± 3.6 LT: 9.1 ± 3.6	RT *p* < 0.05 LT *p* < 0.05
Normalized SUV Cases with RT cortex abnormality (*n* = 75) Cases with LT cortex abnormality (*n* = 54)	Average ± SD RT: 1.22 ± 0.17 LT: 1.18 ± 0.21	Average ± SD RT: 1.29 ± 0.15 LT: 1.27 ± 0.16	RT *p* < 0.05 LT *p* < 0.05
AAI	Average ± SD 7.36 ± 6.83	Average ± SD 3.89 ± 3.06	*p* < 0.00001

**Table 2:** Prevalence of medically refractory disease in patients with and without thalamus hypometabolism.

Patients	Total Cases	Intractable	Percentages	*p*-Value
With thalamus hypometabolism	61	51	83.6%	0.003
Without thalamus hypometabolism	139	90	64.7%	

**Table 3:** Prevalence of no response to surgery in patients with and without thalamus hypometabolism.

Patients	Total Cases	No Response	Percentage	*p*-Value
With thalamus hypometabolism	35	17	48%	0.01
Without thalamus hypometabolism	51	13	25%	

## Data Availability

The datasets used and/or analyzed during the current study are not publicly available due to institutional privacy policies and ethical restrictions related to patient data. However, de-identified data may be made available from the corresponding author upon reasonable request and with appropriate institutional approvals.
